# Is decreased volar tilt and radial length a sign of early rheumatoid arthritis?

**DOI:** 10.1590/1806-9282.20240887

**Published:** 2024-11-22

**Authors:** Elif Altunel Kılınç, Öner Kılınç, Gizem Kırmızıer, Gizem Varkal, İpek Türk, Süleyman Özbek

**Affiliations:** 1 Çukurova University, Faculty of Medicine, Department of Rheumatology – Adana, Turkey.; 2 Mersin City Research and Education Hospital, Department of Orthopedics and Traumatology – Mersin, Turkey.

**Keywords:** Rheumatoid arthritis, X-ray film, Early diagnosis

## Abstract

**OBJECTIVE:**

The aim of our study was to evaluate the effect of anatomical measurements consisting of volar tilt, radial length, radial inclination, and ulnar variance obtained from X-ray radiography in predicting deformity in patients with rheumatoid arthritis and the power of these measurements in predicting rheumatoid arthritis in patients with nonspecific wrist pain.

**METHODS:**

A total of 98 rheumatoid arthritis and 40 control patients presenting with nonspecific wrist pain were cross-sectionally evaluated by X-ray radiography. Rheumatoid arthritis patients were divided into subgroups with and without deformity. Volar tilt, radial length, radial inclination, and ulnar variance measurements were performed.

**RESULTS:**

When the anatomical measurements of rheumatoid arthritis patients with and without wrist deformity were compared with the control group, volar tilt and radial length were significantly lower in rheumatoid arthritis patients with and without wrist deformity than in the control group. There was no difference between rheumatoid arthritis groups with and without deformity.

**CONCLUSIONS:**

Low volar tilt and radial length may be an early X-ray radiographic finding of rheumatoid arthritis and may provide additional diagnostic data in patients presenting with nonspecific wrist pain, especially for seronegative rheumatoid arthritis where diagnosis is difficult.

## INTRODUCTION

Rheumatoid arthritis (RA) is a systemic, autoimmune, inflammatory disease that affects many tissues and organs, including synovial joints and periarticular structures^1^. The most commonly involved joints (70–90%) are the metacarpophalangeal and proximal interphalangeal joints, as well as the wrist^2^. The wrist consists of three joints: distal radioulnar joint, radiocarpal joint, and midcarpal joint^3^.

Posteroanterior (PA) X-ray radiography (conventional radiography) was the first imaging procedure used to evaluate the hand and wrist in RA. Four parameters have been defined to demonstrate the continuity of wrist biomechanics on PA and lateral X-ray radiographs. These are called anatomical measurements and are volar tilt (VT), radial length (RL), radial inclination (RI), and ulnar variance (UV)^4,5^.

- VT: It is the angle between the line joining the anterior and posterior ends of the radius on the lateral radiograph and the perpendicular line drawn to the long axis of the radius. Normal limits are between 0° and 22° with a mean of 11°–12°.
- RL: It is the distance between two parallel lines passing through the tip of the radial styloid and the articular surface of the ulnar head on PA radiography. Normal limits are between 8 and 18 mm, with a mean of 11–12 mm.
- RI angle: It is the angle between the line joining the tip of the radial styloid and the radial part of the distal radioulnar joint and the perpendicular line drawn to the long axis of the radius on the PA radiograph. Normal limits are between 13° and 30°, with a mean of 22°–23°.
- UV: It is the vertical distance between the lunate facet of the radius and the articular surface of the ulnar head. Normally, these two structures are approximately at the same distance, and the normal anatomical variance is 0±2 mm.

Although X-ray radiography was the first imaging modality used for joint evaluation in RA, the information we obtain from this imaging with our current knowledge indicates late-stage disease. However, what is important in RA patients is to predict and prevent permanent deformity before it develops. There are ultrasound and magnetic resonance imaging studies on this subject. However, access to these imaging methods is not always easy and cheap. We think that X-ray radiography, which is the first step among the imaging methods that are constantly developing and the most easily accessible, will provide us with more information than we expect. We hypothesize that changes in VT, RL, RI, and UV measurements may predict deformity in RA patients or may be predictive of future RA development in patients without RA diagnosis but with wrist pain. Our study aims to investigate the role of anatomical measurements (VT, RL, RI, and UV) obtained from X-ray radiography in the development of RA in patients with nonspecific wrist pain and the prediction of wrist deformity in patients with RA.

## METHODS

This cross-sectional study included 98 patients who presented to the rheumatology outpatient clinic of Çukurova University, between June and December 2023, and were diagnosed with RA according to the 2010 American College of Rheumatology criteria, and a control group consisting of 40 gender- and age-matched individuals presenting with nonspecific wrist pain^6^. The study received ethics committee approval from Çukurova University Ethics Committee (5/5/2023-133). Patients who completed part of the study signed an informed consent form.

RA patients who have been diagnosed with RA, are older than 18 years of age, have no history of wrist surgery, do not have another disease affecting the wrist, do not have a serious neurological or psychiatric disease that would prevent them from answering the survey questions, and have a Disease Activity Score (DAS28) less than 5.1 (active period) were included in the study. A total of 98 RA patients were included in the study. They were divided into two subgroups: those with wrist deformity (reduced joint space, ankylosis, and ulnar deviation) and those without. The hospital system provided demographic data (age and gender), laboratory parameters (rheumatoid factor, anti-cyclic citrullinated peptide, erythrocyte sedimentation rate, and C-reactive protein), and posteroanterior and lateral X-ray radiographs for these subgroups. The patients’ disease duration, medications used, occupation, pain status, DAS28, lung involvement, and smoking status were recorded. Patients were given the Patient-Based Wrist Evaluation Questionnaire (PBWEQ)^7^. Öztürk et al. tested the questionnaire's validity and reliability in our country^8^.

The control group included 40 people over the age of 18 years who were not diagnosed with any rheumatological disease, who presented to the outpatient clinic with nonspecific wrist pain, and who had not undergone any hand or wrist surgery.

VT, RL, RI, and UV measurements were obtained using PA and lateral X-ray radiographs. Measurements were performed by the same orthopedist using Picture Archiving and Communicating System viewer v1.0.

### Statistical analysis

The statistical analyses were performed with the aid of the SPSS for the Windows 25.0 software. The variables’ normality was determined using both visual and analytical methods (Kolmogorov-Smirnov/Shapiro-Wilk tests). In the Kolmogorov-Smirnov test, a p-value greater than 0.05 indicated that the data had a normal distribution. When a normal distribution could not be determined, the Mann-Whitney U test was used to compare the patient and control groups. For group comparisons of qualitative variables, the chi-square test was used. The receiver operating characteristic (ROC) curve was used to compare the area under the curve (AUC), sensitivity, specificity, and cut-off values. The Spearman correlation test was used to evaluate the correlation, and the correlation coefficient was designated as rho. The correlation coefficient of 0.25 indicates no or very weak relationship, 0.25–0.5 indicates a weak-moderate relationship, 0.5–0.75 indicates a strong relationship, and >0.75 indicates a very strong relationship. To assess the diagnostic power of the measurement parameters, a logistic regression analysis was performed. Statistically, p<0.05 was used.

## RESULTS

The study included 98 RA patients (78.5% female; mean age: 56.45±13.32 years) and 40 controls (80% female; mean age: 53.63±11.19 years). A total of 47 patients (82.9% female; mean age: 59.8±14.3 years) in the RA group had wrist deformities, while 51 patients (74.5% female; mean age: 53.2±11.5 years) had no deformities. The gender and age distributions of the patient and control groups did not differ statistically (Table 1).

**Table 1:** Characteristics and demographic data of rheumatoid arthritis and control group.

		Rheumatoid arthritis		Control group	p-value
		With deformity	Without deformity		
Gender	Female (n)	39	38	32	0.310
	Male (n)	8	13	8	
Age±SD		59.8±14.3	53.2±11.5	53.63±11.1	0.130
Extraarticular involvement, n (%)		19 (40.4)	14 (27.4)	-	0.177
DAS28, mean±SD		3.3±1.4	2.5±1.08	-	0.013
Wrist evaluation scores, mean±SD		70±31.5	51±28.6	-	0.001*
Cigarette, n (%)		12 (25.5)	16 (31.3)	-	0.488
Seropositive, n (%)		35 (74.4)	40 (78.4)	-	0.645
Disease duration (month±SD)		133.87±66.9	88.8±59.5	-	0.001*

DAS28: Disease Activity Score; n: count; SD: standard deviation.

* p<0.05.

There was no significant difference in extra-articular involvement, DAS28, smoking history/use, or seropositivity between RA patients with and without wrist deformity (p=0.177, p=0.13, p=0.488, and p=0.645). PBWEQ results were significantly higher in the RA patients with wrist deformity than in the RA patients without wrist deformity (p=0.001; Table 1). Anatomical measurements were evaluated according to disease year. There was a positive, weak-moderate correlation between wrist deformity and disease year (r=0.328, p=0.001).

The RA patients were divided with and without wrist deformity, and the control group was analyzed into three subgroups. When RA patients with wrist deformity were compared to the control group, VT and RL were significantly lower in the RA patients with wrist deformity group. The RI and UV measurements did not differ significantly (Table 2). When RA patients with wrist deformity were compared to RA patients without deformity, there were no significant differences in the measurements of VT, RL, RI, and UV. When RA patients without wrist deformity were compared with the control group, VT and RL were significantly lower, while RI and UV measurements were not statistically significant (Table 2).

**Table 2:** Evaluation of functional and X-ray measurements of the groups.

		RA with wrist deformity	RA without wrist deformity	Control group	p-value
Anatomic measurements	VT mean±SD	3.3±4.4	3.7±4.4	10.2±5.4	<0.05*
	RL mean±SD	7.6±2.1	8.1±2.1	10.8±2.5	<0.05*
	Rİ mean±SD	20.5±4.67	20.94±3.8	21±2.5	0.787
	UV mean±SD	1.1±3.1	0.29±1.4	0.022±1.1	0.193

Rİ: radial inclination; RL: radial length; SD: standard deviation; UDA: ulnar deviation angle; UV: ulnar variance; VT: volar tilt; RA: rheumatoid arthritis.

* p<0.05.

To evaluate the anatomical measurements, ROC analysis was performed in RA patients with and without deformity. The analysis revealed that the power of VT and RL to detect deformity was not significant (p=0.078 and p=0.860, respectively). When the RA without deformity group and the control group were evaluated by ROC analysis, the power of VT and RL to distinguish RA without deformity from the control group was statistically significant (p<0.05 for all). Cut-off values were calculated as 7.9 for VT and 9.1 for RL. AUC (95%CI) was 0.862 (0.768–0.956) for VT and 0.791 (0.7–0.882) for RL. According to the result of ROC analysis between RA without deformity and the control group, the positive predictive rate of VT was 86%, while the positive predictive value of RL was 66%. When all RA patients and controls were evaluated, the positive predictive values of VT and RL were 94 and 88%, respectively. Whether these measurements have diagnostic value for RA is worth investigating.

In the multivariate reduced model, an independent effect of VT and RL in predicting RA was observed (p<0.05 for all). The prediction accuracy rate for VT alone was 91.3%. The prediction accuracy rate is 90.6% with the combined evaluation of VT and RL. When RA without deformity and the control group were evaluated, the independent effect of VT and RL was similarly observed (p<0.05 for all). The prediction accuracy rate for VT alone was 85.6%. When VT and RL were evaluated together, the prediction accuracy rate was 87.8%. The results are given in Table 3.

**Table 3:** Regression analysis results of volar tilt and radial length.

	All RA and control group		RA without deformity and control group	
	VT	RL	VT	RL
B	0.330	0.619	0.283	0.544
SE	0.65	0.129	0.071	0.15
OR (95%CI)	1.4 (1.232–1.590)	1.85 (1.441–2.392)	1.3 (1.15–1.52)	1.7 (1.3–2.9)
p-value	<0.05	<0.05	<0.05	<0.05

B: regression coefficient; RL: radial length; SE: standard error; OR: odds ratio; VT: volar tilt; RA: rheumatoid arthritis; CI: confidence interval.

## DISCUSSION

In our study, anatomical measurements were made using X-ray radiography in RA patients and patients presenting with nonspecific wrist pain. According to the results of our study, anatomical measurements did not predict deformity in RA patients. However, VT and RL were significantly lower in RA patients with and without wrist deformity compared to the control group. ROC analysis revealed the power of VT and RL in distinguishing the RA group without deformity from the control group. The fact that VT and RL were significantly lower in RA patients than in patients with nonspecific wrist pain suggested that these angles may be a supportive finding when diagnosing RA and that these angles may have been affected in the early period before diagnosis. To the best of our knowledge, our study is the first study in which anatomical measurements were evaluated in RA patients.

As it is known, RA most commonly affects the hand and wrist joints, causing permanent deformities^9^. Therefore, one of the primary goals in RA patients is to prevent the development of deformity and preserve the functionality of the wrist, which is one of the most important joints for daily activities. Some studies evaluating the postoperative functionality of distal radius fractures with anatomical measurements suggest that the decrease in RL and VT is associated with poor functional outcomes^7,10-12^. However, many orthopedic studies showed that normal VT should be maintained in terms of carpal bone alignment and wrist functions^13,14^. In our study, RL and VT in RA patients with and without wrist deformity were significantly lower than in the control group. There was no difference between RA patient groups with and without wrist deformity. The fact that RL was lower in the group with wrist deformity compared to other groups was an expected result due to the effect on functionality. However, although there was no loss of function in the group without wrist deformity, it was lower than the control group. This shows that the decrease in RL and VT has an undesirable effect on wrist function and does not simply mean loss of function. Based on our statistical analysis, we interpret low RL and VT as measures that are predictive of RA or affected in the early stage of RA.

In a study evaluating adequate and inadequate reduction in the postoperative period after distal radius fracture, it was reported that RI was significantly lower in the inadequate reduction group^11^. When all groups were evaluated in our study, no significant difference was detected between RI values. In our study, although RI did not change between groups, there was a decrease in RL. Wrist involvement in RA affects the bone metaphysis, and this situation shows that it affects the length without changing the angle.

In a study conducted on patients who underwent reduction with volar plating after distal radius fracture, it was found that PBWEQ scores showed a negative correlation with functional measurements measured with a goniometer^11^. Although functional measurements were not examined in our study, no correlation was detected when anatomical measurements were examined. There is no other study in the literature that supports these results.

The limitation of our study is that it is a cross-sectional case-control study. In our study, decreased VT and RL were concluded to be predictive of RA or early-stage radiologic changes of RA. However, since the study was a cross-sectional study, it is not possible to say this result with certainty. Evaluation of anatomical measurements with X-ray radiography in patients with nonspecific wrist pain and prospective evaluation in terms of RA development will strengthen this hypothesis. On the contrary, although the early-stage findings of RA patients have been evaluated with many magnetic resonance imaging and ultrasound studies, our study is one of the rare studies on X-ray radiography, which we use in the first step. Supporting this hypothesis with prospective studies will increase the clinical importance of X-ray radiography, which is an easily accessible and inexpensive method.

## CONCLUSION

Our study found that VT and RL were statistically significantly lower in the RA group without deformity compared to the control group. In addition, VT and RL were found to be independent effect factors in the RA group without deformity and statistically significant in differentiating from the control group. Based on all these results, we concluded that low VT and RL may be affected in the early stages of RA and that measurements evaluated by X-ray, which is used as the first imaging method in patients presenting with wrist pain, may provide additional diagnostic data, especially in seronegative RA patients with difficult diagnosis.
